# Rheological characteristics of faecal sludge from VIP latrines and implications on pit emptying

**DOI:** 10.1016/j.jenvman.2018.08.098

**Published:** 2018-12-15

**Authors:** S. Septien, J. Pocock, L. Teba, K. Velkushanova, C.A. Buckley

**Affiliations:** a Pollution Research Group, University of KwaZulu-Natal, Howard College, 4001, Durban, South Africa; b Chemical Engineering, University of KwaZulu-Natal, Howard College, 4001, Durban, South Africa

**Keywords:** Faecal sludge, VIP latrines, Pit emptying rheology, Shear-thinning, Yield stress

## Abstract

This work aims at characterizing the rheological properties of faecal sludge from Ventilated Improved Pit (VIP) latrines and their implication on pit emptying. Faecal sludge was sampled from 3 pit latrines located in the eThekwini Municipality (Durban, South Africa). Samples were taken at different positions within the pit. For each of the samples, measurements in the rheometer in triplicates were performed in order to determine their rheological properties, and their moisture and ash content were measured also in triplicates. Experiments in the rheometer were performed for samples for which its moisture content was modified. In order to better understand the influence of water addition into the pit. During pit emptying, calculations were carried out from the experimental data, based in the criteria set in the Omni-Ingestor initiative, carried out by the Bill & Melinda Gates Foundation.

Faecal sludge exhibited a shear thinning behaviour, i.e. a decrease in viscosity with increasing shear rate, and presented a yield stress comprised between 500 to 1000 Pa. This needs to be surpassed in order to overcome the elastic resistance of the sludge to flow. Similar viscosities were found for the samples from the different pits, irrespective of the position within the pit, except for the sample from the bottom of one of the pits for which it was not possible to induce a flow. This sample had a considerably lower moisture content (67% wet basis) compared to the other samples (around 80% wet basis), probably due to a higher biodegradation as it was the most aged sludge in the pit.

According to the experimental results and calculations, the pumping requirements during pit emptying will decrease drastically by increasing the moisture content of the sludge. The addition of water into the pit would then facilitate the pit emptying operation by reducing the head and power required for pumping. However, this practice would require employing considerable amounts of water and handling higher volumes of sludge, which would lead to longer pit emptying times and increase the difficulty of the operation. For example, increasing the moisture content of the sludge from 75 to 90% will reduce the head and power of the pump by a factor 100, but will triplicate the amount of water in the sludge and, consequently, the time for pit emptying. Therefore, a compromise has to be made between increasing the pumping feasibility and adding water to the pit.

## Highlights

• The rheological properties of faecal sludge from pit latrines were characterized.
• Faecal sludge showed a shear thinning behaviour with a yield stress.
• Faecal sludge rheological characteristics were similar at different pit positions.
• The rheological characteristics were similar for sludge from different pit latrines.
• Adding water in the sludge decreases its viscosity and facilitates pit emptying.

## 1 Introduction

Pit latrines are the predominant form of sanitation in low-income countries, particularly in Africa, because of their low-cost (Cairncross et al., 2010, Habitat, 2008, Jain, 2011). The main inconvenience with this type of sanitation facility is that the pit eventually fills up and thus the latrine cannot be used any further (Strauss et al., 1999, Thye et al., 2011). This causes a major problem in densely populated areas, as there is a lack of space for the relocation of the toilets, whilst the accumulation of high amounts of faecal sludge can lead to the spread of waterborne diseases and pollution of the environment. Hence, the safe emptying of the full pit is a necessity for sustainable faecal sludge management. The collected sludge has to be treated for pathogen inactivation and resource recovery, such as water for reuse, compost, fertilizer, biofuel, chemicals and construction materials (Dodane and Ronteltap, 2014).

Safely organized pit emptying, transportation and processing of faecal sludge are crucial for the sustainable management of pit latrines. Technological solutions have been developed in order to overcome the challenges related to the handling of faecal sludge, which is a biohazardous material with potentially high pathogen content. Pit emptying devices have been designed in order to avoid traditional manual emptying practices where the worker is exposed to a high biological contamination risk (Mikhael et al., 2014, O'Riordan, 2009). These pit emptying technologies include pumping systems (among diaphragm pumps, trash pumps, Gobbler), pit screw augers and vacuum systems (Mikhael et al., 2014, O'Riordan, 2009). Efforts have being made in order to provide improved technical solutions for pit emptying within the Omni-Ingestor (OI) project (Strande, 2014), funded by the Bill & Melinda Gates Foundation (BMGF). The initiative Sanitation Fund Research for Africa, funded by the BMGF and the Water Research Commission from South Africa, places particular emphasis on the development of innovative techniques for pit desludging in Sub-Saharan African countries (Pillay and Bhagwan, 2014).

The rheology of faecal sludge is an important parameter for the design and operation of pit emptying devices, as well as certain treatment process technologies, such as the viscous heater that pasteurizes the sludge after heating the material by viscous friction (Belcher et al., 2015). Currently, there is no enough information in literature on the rheological characteristics of faecal sludge. Only a few papers from the same authors deal with the rheology of human faecal material (Woolley et al., 2014b, Woolley et al., 2014a). These papers focused on the study and modelling of the change of viscosity of fresh faeces with shear rate as a function of the moisture content and temperature. The results highlighted the shear-thinning and thixotropic behaviour of the faecal material, which was also observed for other types of slurry material such as sewage sludge and cattle manure (Amiri et al., 2012, El-Mashad et al., 2005, Ghafoori et al., 2007, Seyssiecq et al., 2003). In the study of Woolley et al., 2014a, Woolley et al., 2014b, fresh faeces also exhibited a decrease of viscosity by increasing the temperature or the faeces moisture content. Like this, the viscosity decreased from 5 to 1 Pa s by increasing the moisture content from 67.5 to 85% at a shear rate of 100 s^−1^. Increasing the temperature of the sludge from 10 to 50 °C reduced the viscosity from 450 to 275 Pa.s, at a shear rate of 1 s^−1^.

Nevertheless, the characteristics of fresh faeces, manure and sewage sludge differ to those of faecal sludge from pit latrines, in properties such as the moisture content, aging and degradability degree, which should lead to a different rheological behaviour. Zuma et al. (2015) and Bakare et al. (2012) found that the physico-chemical properties of faecal sludge can vary as a function of the latrine and position within the pit. Moreover, as pointed out by Niwagaba et al. (2014), the properties of faecal sludge can differ largely from one sanitation facility to another, due to diverse factors such as the local diet, disposal of trash and grey water, water infiltration and drainage of leachate. Biodegradation occurs within the pit and the degree of biodegradation depends on the age of the faecal sludge (Nwaneri, 2009, Nwaneri et al., 2008). In fact, the most aged faecal sludge situated at the bottom of the pit tends to be more biodegraded than the fresh sludge layers above it. Environmental conditions, such as temperature or water from rainfall that can be infiltrated into the pit, can affect the properties of the sludge. This variability could then have an influence on the rheological characteristics of the sludge.

The present work was performed under the context of the “Reinvent The Toilet Challenge” program, initiated by the BMGF in order to improve the access to sanitation of people living in poverty (BMGF, 2012). This study aims to fill in the gap of knowledge on the rheological characteristics of faecal sludge, and to provide useful data to researchers and sanitation practitioners for the design of pit emptying equipment. The rheology of faecal sludge is an important of ventilated improved pit (VIP) latrines from the eThekwini municipality were characterized. Additional experiments were conducted by increasing the moisture content of faecal sludge, in order to simulate the material obtained from common pit emptying practices where water is added to the sludge in order to increase its ability to be pumped. From the experimental data, calculations were performed in order to better understand the influence of the sludge moisture content during pit emptying. The effect of temperature on the rheological characteristics was not included in this work, as the temperature in the laboratory during the experiments, namely 20 °C, was similar to that in the eThekwini municipality, varying in average between 18 and 25 °C along the year.

Note that the term “feacal sludge” employed in this study denotes the waste from VIP latrines whereas “fresh faeces” refers to the waste dealt in the investigation of Woolley et al., 2014a, Woolley et al., 2014b.

## 2 Material and methods

### 2.1 Feedstock: faecal sludge from VIP latrines

The feedstock in this study was faecal sludge of VIP latrines from the eThekwini Municipality (Durban metropolis, South Africa). This type of on-site sanitation facility was designed in order to improve conventional pit latrine by reducing odours and the number of flies within the toilet. The VIP latrines consist of an enclosed brick superstructure with a door, a pit with a concrete slab cover and a ventilation pipe that pulls out the malodorous. The pipe is installed with a fly screen at the top, which prevents flies from entering the latrine.

#### 2.1.1 Collection

The VIP latrines selected for sampling were from different locations in the peri-urban area from the eThekwini municipality but the socio-economic environment was the same between the different cases. For practical reasons, the sludge collected from only three VIP latrines were included in this study, although a considerably more important number of pits would be suitable in order to have a better statistical representation of the population. Nonetheless, the sludge from the three pits did not exhibit any visible peculiarity and presented characteristics that were representative of the faecal sludge found typically in the VIP latrines from the municipality, as demonstrated in section 4.1.

**Fig. 1:**
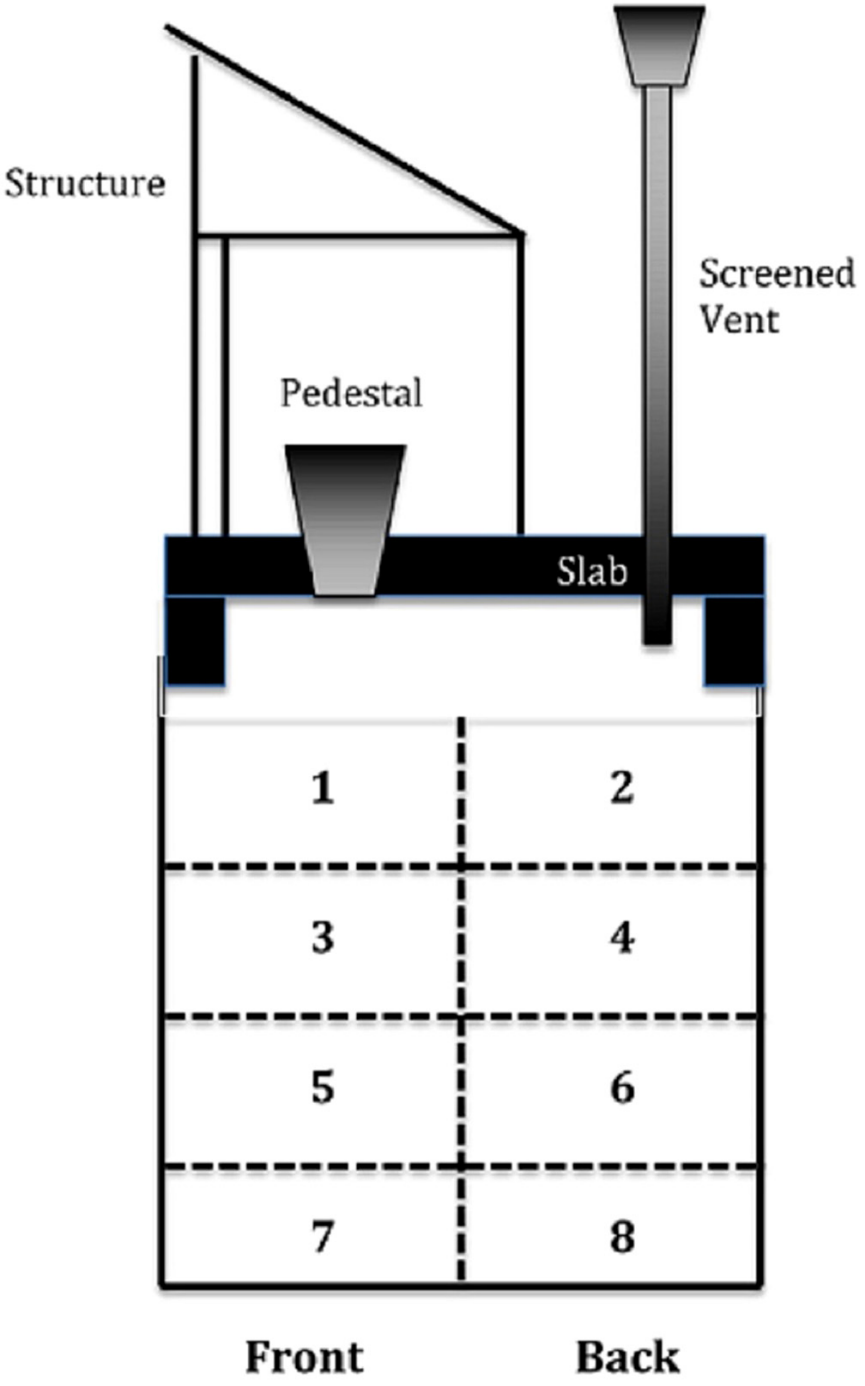
Scheme of the different sampling sections in the VIP latrine, after Zuma et al. (2015).

Faecal sludge was sampled from different sections of the pit. Sampling was performed according to a diagram proposed by Zuma et al. (2015), where the pit is divided vertically into a back and front section, and four horizontal layers, as depicted in Fig. 1. Note that the age of the sludge increases from section 1–8. This division of samples was done for two pits (pit 1 and 2). For a third pit (pit 3), the samples from the different sections were mixed in order to give an average representation of the content. The amount of sludge collected per sample was of a few litres.

**Fig. 2:**
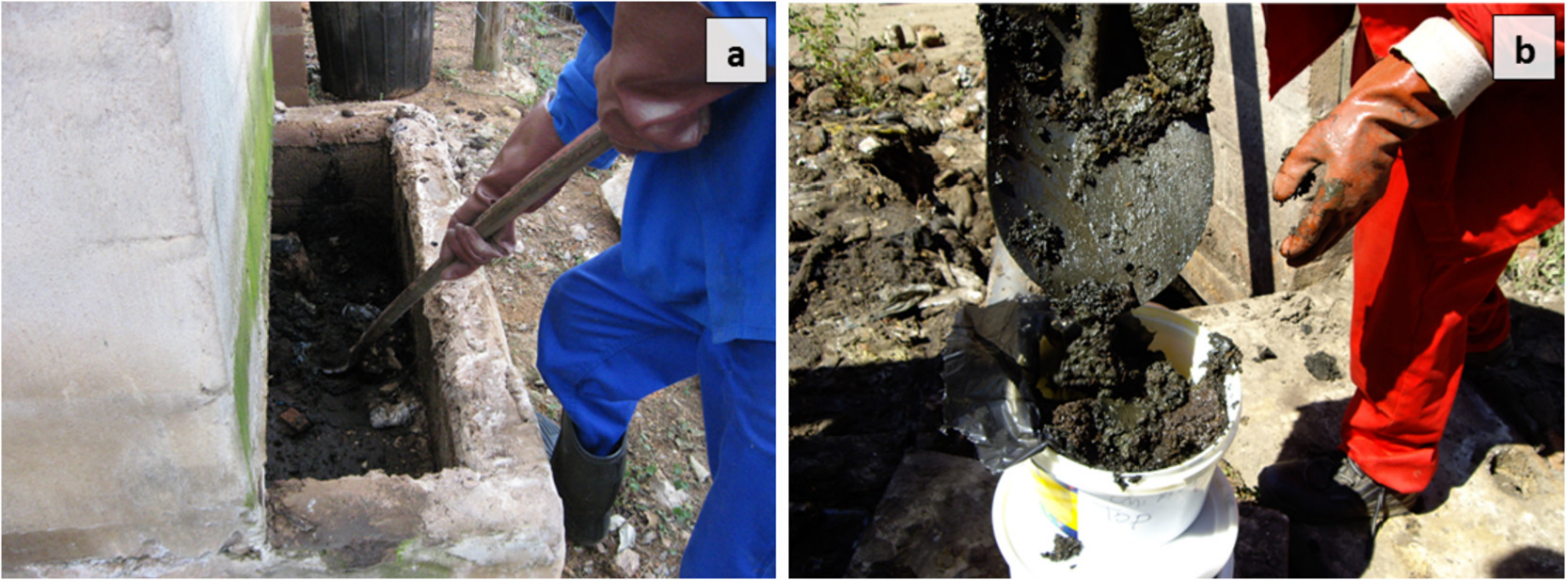
Procedures of pit emptying in the eThekwini municipality (a) and sampling (b).

In the eThekwini municipality, the VIP latrines are usually emptied in a 5-year cycle (Zuma et al., 2015), so it can be assumed that the age of the sludge was of 5 years at the bottom of the pit while it was more recent at the top, with an intermediary age between the bottom and top layers. The average age of the sludge can be then assumed to be 2.5 years. Pit emptying was performed manually without water addition, by using tools as shovels for digging and pitchfork to remove the big pieces of trash. Sampling was done using a spatula to put the sludge into containers for its transportation. During these operations, faecal sludge did not undergo considerable shear stress, so the rheological properties can be considered not have been significantly affected. Fig. 2 displays how pit emptying and sampling proceeded.

#### 2.1.2 Storage

After collection, the samples were transported to the laboratory of the Pollution Research Group and stored in a cold room at 4 °C in order to preserve their properties. The samples were kept in closed containers in order to avoid desiccation. Non-homogeneous debris larger than 5 mm were selectively removed through sieving.

### 2.2 Analytical test

Different analytical tests were performed on the faecal sludge samples using the Standard Operation Procedures from the Pollution Research Group (PRG, 2014).

#### 2.2.1 Moisture and ash analysis

The moisture and ash content were measured in triplicates for each sample, based on the Standards Methods for the Examination of Water and Wastewater (APHA, 2012). The moisture content of the samples was determined by measuring the sample mass before and after complete drying in an oven at 105 °C for 24 h. After drying, the ash content was determined by measuring the mass before and after calcination in a muffle furnace at 550 °C for 2 h.

The moisture and ash content measurements were done in triplicates for each sample. The uncertainty of these measurements was determined by applying a t-student distribution with an interval confidence of 90%.

#### 2.2.2 Rheological analysis

The rheological properties of the samples were measured by a rheometer model *Anton Paar MCR 51*, at laboratory temperature (20 °C). During the experiments, the measurements were performed through the rotation of a vane within the sludge that was contained inside a cup. The shear stress and viscosity were determined by the software of the analyser, through the measurement of the torque from the rotation of the vane. The tests were performed in the samples at increasing rotational shear rate in the range 0.001–1000 s^−1^. The total time of an analysis varied between 20 and 30 min. Each experiment was repeated twice or three times using a different sub-sample from the same sample. The results were verified to be repeatable.

Analyses in the rheometer were also performed after varying the moisture content of the sample from pit 3. The average moisture content of raw faecal sludge was 80%, corresponding to 4 g moisture/g dry solid. This was increased to 81, 84, 87 and 90% (4.2, 5.3, 6.7 and 9.0 g moisture/g dry solid respectively) by the addition of water, and decreased to 77% (3.3 g moisture/g dry solid) after mixing raw and oven dried sludge.

It has to be noted that the phenomenon of wall-slip could influence the measurements during analysis in the rheometer. The results obtained could be then considered as “apparent” and not as “true”. Nevertheless, if a wall-slip effects existed between the sludge and the walls of the cup, the comparison of the results from the different samples between themselves is still valid. The presence of a wall-slip effect has to be investigated in future works.

### 2.3 Calculations of the pumping requirements during pit emptying

Calculations were performed in order to better understand the influence of the moisture content of the sludge during pit emptying operations. Note that some of the calculation parameters were obtained from experimental data issued from the measurements in the rheometer, whereas the flow geometry is different in a pipe. Therefore, the results from the calculations may deviate from reality, and they cannot be employed for design or operation proposes. Indeed, the calculations only provide indicative trends and order of magnitude to take into consideration.

The calculations were based on the criteria set in the OI project workshop:

i) a pit emptying rate set at a value of 3 L/s for an efficient operation;
ii) tank inlet at aperture of 0.2 m in order to limit blockages from the trash disposed in the pit;
iii) hose horizontal length of 100 m in order to have access to remote pits.

From the assumptions above, the average velocity of the sludge in the pipe was approximately 0.01 m s^−1^, leading to a shear rate of 0.5 s^−1^. For this exercise, the moisture content of the faecal sludge was varied in order to simulate the pit emptying practices where water is added to the pit. For simplification purposes, the terrain was considered as flat and the lift of sludge during the operation was neglected; the efficiency of the pump was assumed to be close to one.

The head and power for pumping during pit emptying were calculated from the following methodology that considers the flow of a non-Newtonian fluid within a pipe (Brown and Heywood, 1991, Chilton and Stainsby, 1998, Metzner and Reed, 1955):

i. Calculation of the power law Reynolds number through Equation (1)

(1) $$Re^{'}=\frac{VD\rho_{m}}{K}{\left(\frac{4n}{1+3n}\right)}^{n}{\left(\frac{D}{8V}\right)}^{n-1}$$

ii. Calculation of the power law friction factor through Equation (2)

(2) $$f=\frac{16}{Re^{'}}$$

iii. Calculation of the drop of pressure in a pipe through Equation (3)

(3) $$\Delta P=\frac{4\cdot \rho_{m}\cdot f\cdot L\cdot V^{2}}{2\cdot D}$$

iv. Calculation of the friction head losses through Equation (4)

(4) $$H=\frac{\Delta P}{\rho_{m}\cdot g}$$

v. Calculation of the hydraulic power through Equation (5)

(5) $$P=H\cdot \rho_{m}\cdot g\cdot Q$$

Where: Q is the flowrate of the sludge during pit emptying (m^3^·s^−1^); V is the average velocity of the sludge (m·s^−1^); D and L are respectively the diameter and length of the pipe (m); $\rho_{m}$ is the density of the sludge (kg·m^−3^); K is the consistency coefficient (−); n is the flow behaviour index (−); f is the friction factor (−); $Re^{'}$ is the power law fluid modified Reynolds number (−); g is the standard gravity (9.81 m.s^−2^); ΔP is the drop of pressure in the pipe (Pa); H is the frictional head losses by the pump; P is the hydraulic power (W). Note that the viscosity term in the Reynolds number in Equation (1) has been substituted by its expression that takes into account its variation as a function of the shear rate.

The parameters *K* and *n* can be determined through the experimental data using the power law model (Equation (6)). For this, the shear stress was regressed versus the shear rate for the samples with varying moisture content (77, 81, 84, 87 and 90%). The regression was done in a shear rate range of 0.01–100 s^−1^, as the shear rate in our application was comprised in this range and the fitting using the power law was the highest (R^2^ > 0.95). Thereafter, the values obtained for *K* and *n* were correlated to the moisture content of the samples, as displayed in Equations (7), (8). The correlation of *K* versus moisture content led to a coefficient of determination *R*^*2*^ of 0.995, indicating an accurate fitting, whilst the relationship of *n* with moisture content gave a *R*^*2*^ value of 0.818, indicating a moderate fitting.

(6) $$\tau =K\cdot {\dot{\gamma }}^{n}$$

(7) $$K=0.59\times {\left(MC\right)}^{-30}$$

(8) $$n=1.2\times {\left(MC\right)}^{13.5}$$

Where: τ is the shear stress (Pa); $\dot{\gamma }$ is the shear rate (s^−1^); *MC* is the moisture content in wet basis.

## 3 Results and discussion

### 3.1 Effect of moisture content on the rheological properties of faecal sludge

This section describes the results obtained in the rheometer for the samples whose moisture content was altered by the addition of water or dried material. In particular, it discusses the effect of moisture content on the viscosity and yield stress of the sludge.

#### 3.1.1 Effect on the viscosity of the sludge

**Fig. 3:**
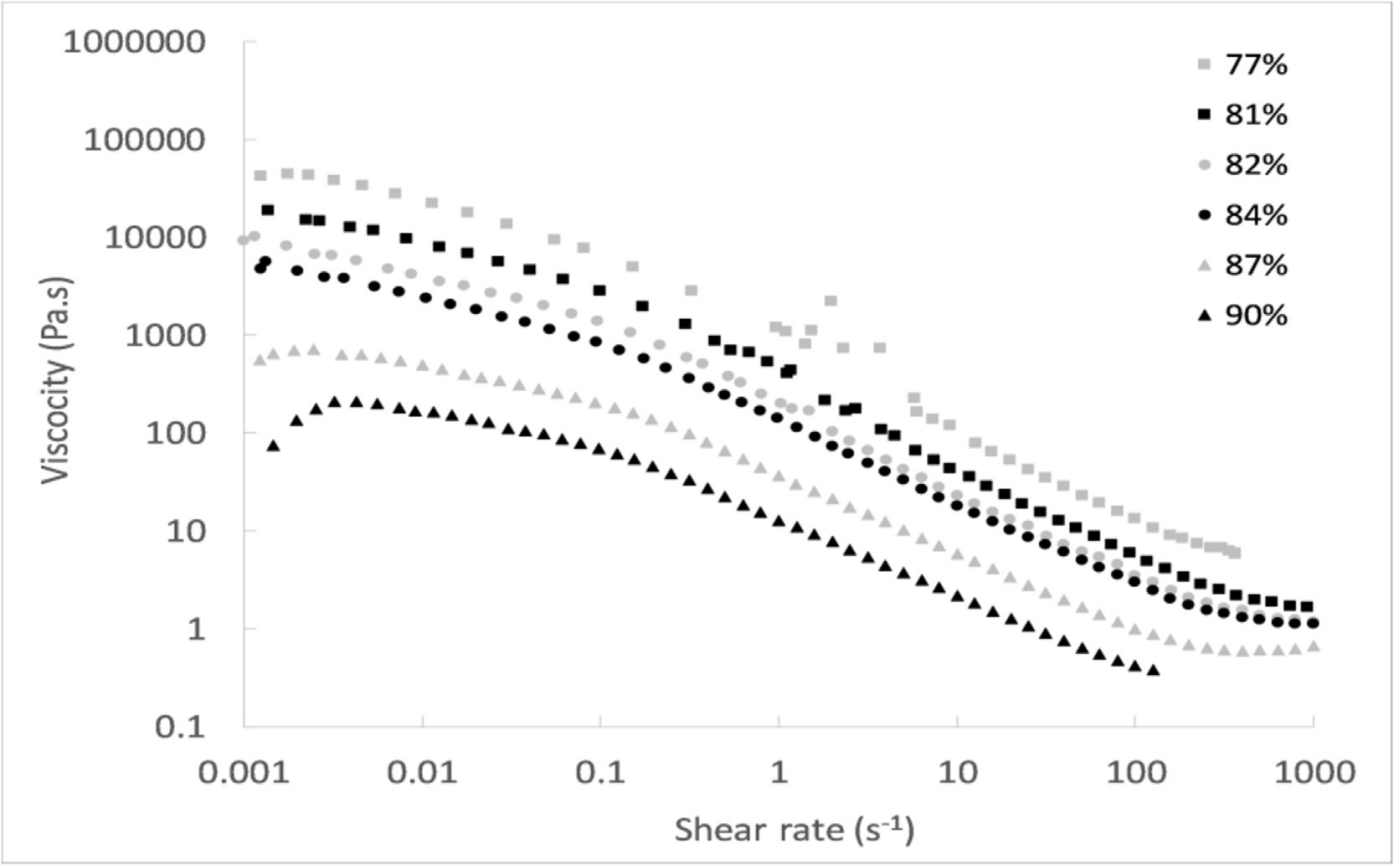
Viscosity versus shear rate for faecal sludge with different moisture contents.

Fig. 3 displays the viscosity of the samples with a moisture content from 77 to 90%, as a function of the shear rate. It can be seen that all the viscosity curves followed the pattern of shear thinning fluid, i.e. decrease of viscosity by increasing the shear rate. The viscosity decreased as the moisture content was higher, as also observed by Woolley et al. (2014b). At a shear rate of 0.1 s^−1^, the sludge with a moisture content of 81–82% exhibited a viscosity near to 1000 Pa s^−1^. The reduction of its moisture content to 77% increased its viscosity of one order of magnitude, while the viscosity dropped of one order of magnitude by increasing its moisture content up to 87–90%. At a shear rate of 100 s^−1^, the sample with moisture content of 77% had a viscosity of 10 Pa s^−1^, while the increase of moisture content above 87% led to a reduction of viscosity below 1 Pa s^−1^.

As it could be expected, the increase of the shear stress and moisture content enabled to reduce the viscosity of faecal sludge in a significant way. This is beneficial for pit emptying, as less effort would be required to pump the sludge from the pit.

#### 3.1.2 Effect on the yield stress

**Fig. 4:**
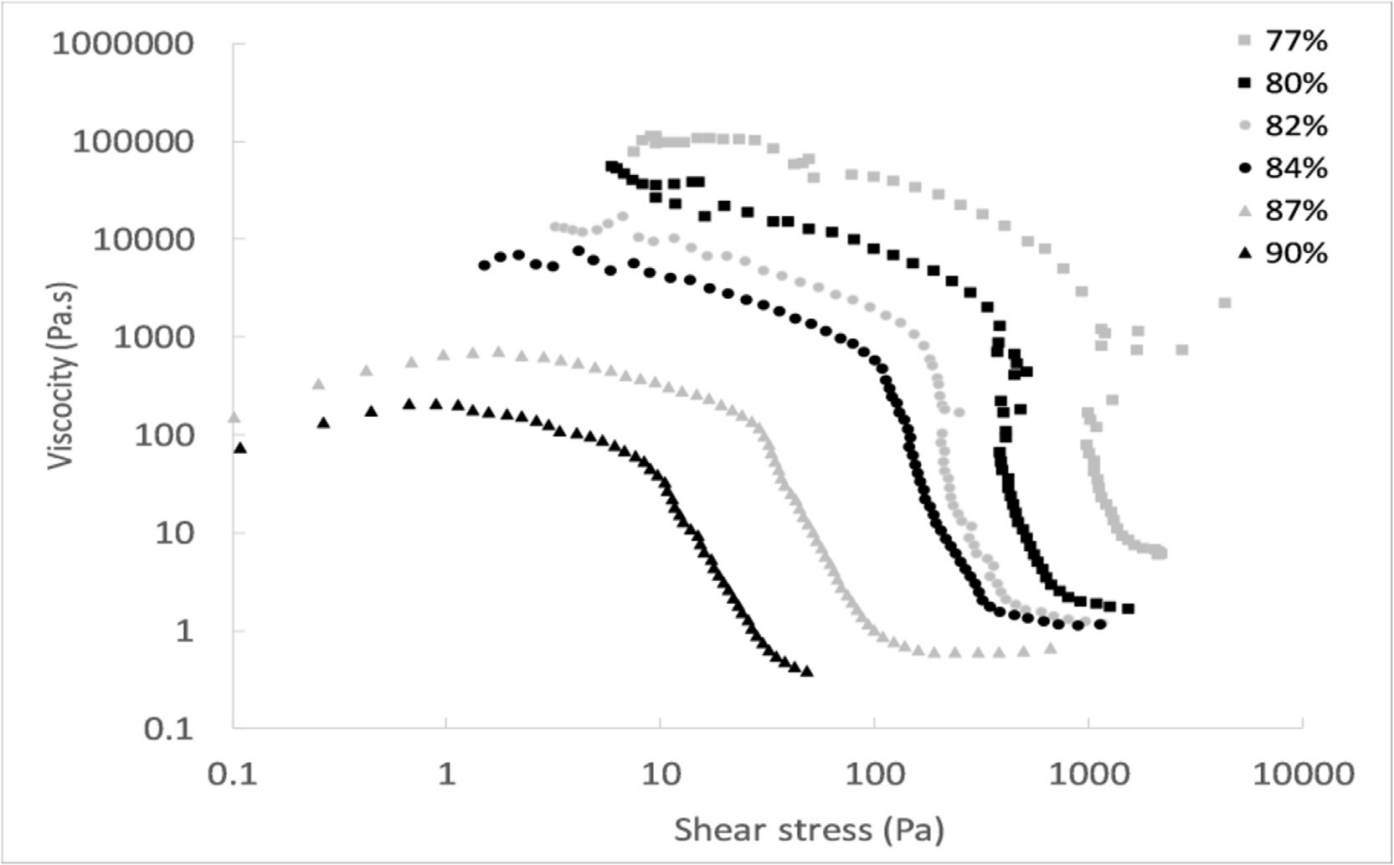
Viscosity versus shear stress for faecal sludge with different moisture contents.

Fig. 4 displays the viscosity versus shear stress for the samples with different moisture contents. All the curves exhibited a similar shape. As the shear stress increased, the viscosity declined smoothly until reaching a point from which it fell of a few orders of magnitude. This breakpoint corresponds to the yield stress of the sludge, from which it started to deform in a plastic way instead in an elastic manner. The yield stress is the minimum shear stress value that has to be reached in order to overcome the elastic resistance of the sludge and induce a flow.

**Fig. 5:**
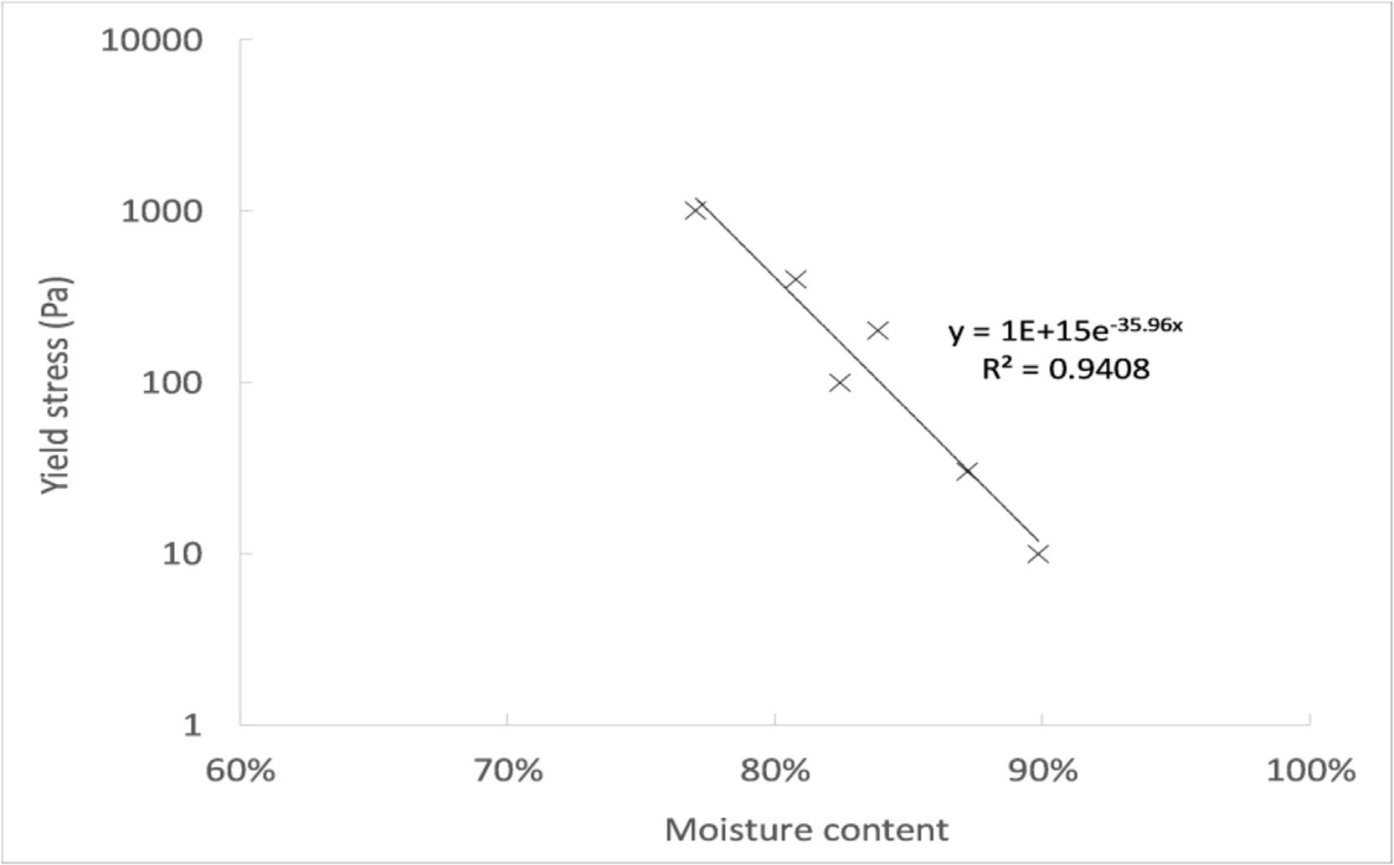
Yield stress as a function of the faecal sludge moisture content.

Fig. 5 shown the evolution of the yield stress as a function of the moisture content of the sludge. It can be seen that the yield stress tended to decrease at higher moisture content. Like this, the yield stress diminished from 1000–10 Pa by increasing the moisture content from 77 to 90%, which represents a difference of two orders of magnitude. This pattern followed an exponential evolution, as suggested by the trendline in Fig. 5.

The increase of the moisture content enabled to lower the yield stress of the sludge, which has positive repercussions on pit emptying. This way, the elastic resistance of the sludge to flow can be reduced, which facilitates to initiate a flow during pumping.

### 3.2 Comparison of rheological properties of the faecal sludge samples

This section compares the rheological properties of faecal sludge sampled from different positions within a pit and between different pits.

#### 3.2.1 Composition of the samples

**Fig. 6:**
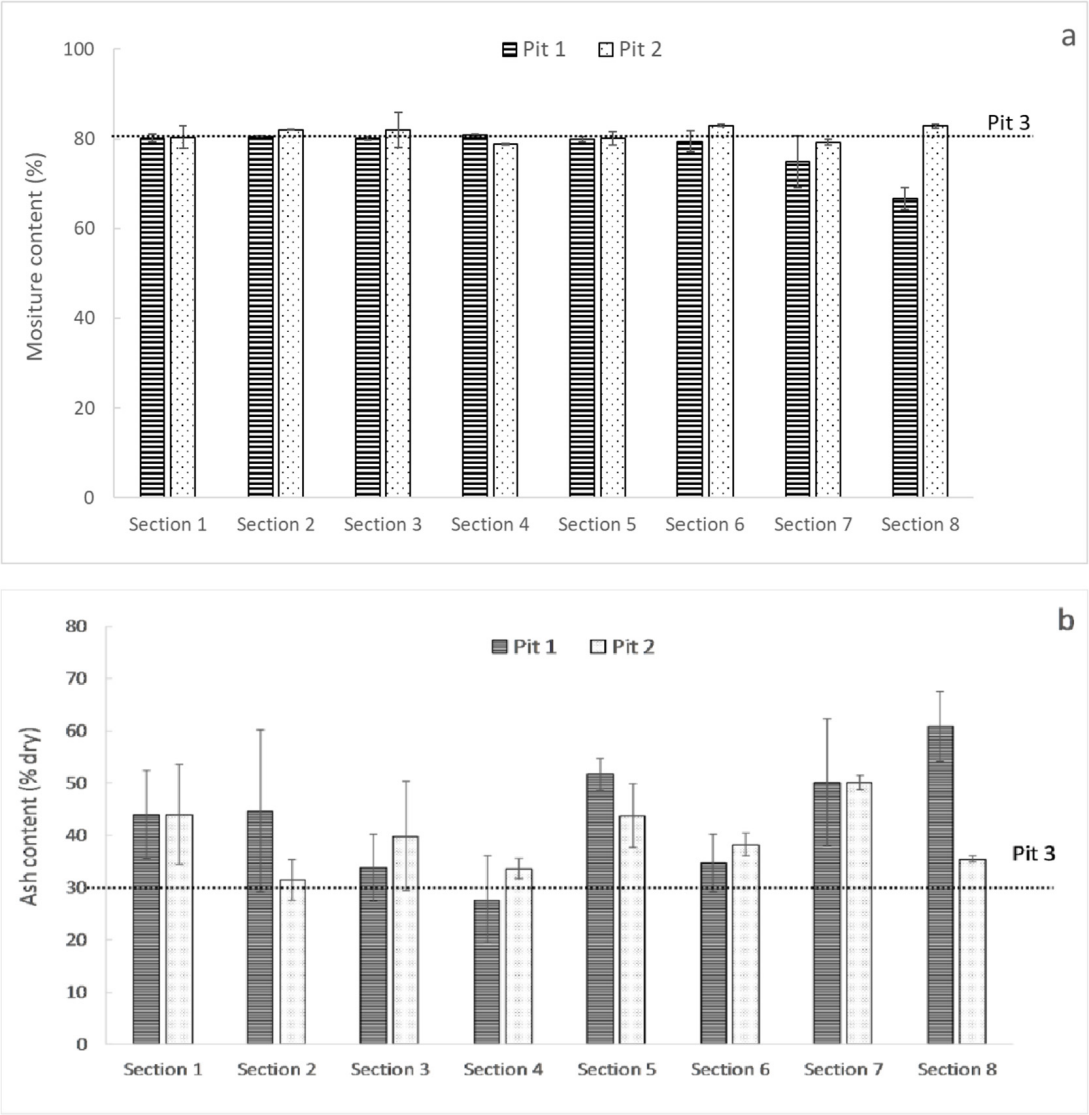
Moisture content (a) and ash content (b) of the samples from pit 1, 2 and 3.

Fig. 6 presents the distribution of the moisture and ash content within the different sections of pit 1 and 2, and the average moisture content of pit 3.

As seen in Fig. 6a, the samples presented a similar moisture content of about 80% (corresponding to 4.0 g moisture/g dry solid). The only exception was the sample from section 8 of pit 1, which exhibited a considerable lower moisture content of about 67% (2.0 g moisture/g dry solid). As possible explanation, this sample, which was the most aged in pit 1, could have undergone a higher biodegradation compared to the other sections, provoking a lower moisture content. An alternative explanation could be the loss of moisture due to the drainage of leachate through the porous pit base.

Most of the samples had an ash content that varied between 30 and 60% in dry basis, without showing any particular trend due to the high uncertainty bars (Fig. 6b).

The moisture and ash content of the samples were close to the median values reported in the eThekwini municipality (Zuma et al., 2015), which were 81 and 40% respectively. This result suggests that the faecal sludge from this work represented well the sludge typically found in the VIP latrines from the municipality.

#### 3.2.2 Comparison as a function of the position within the pit

**Fig. 7:**
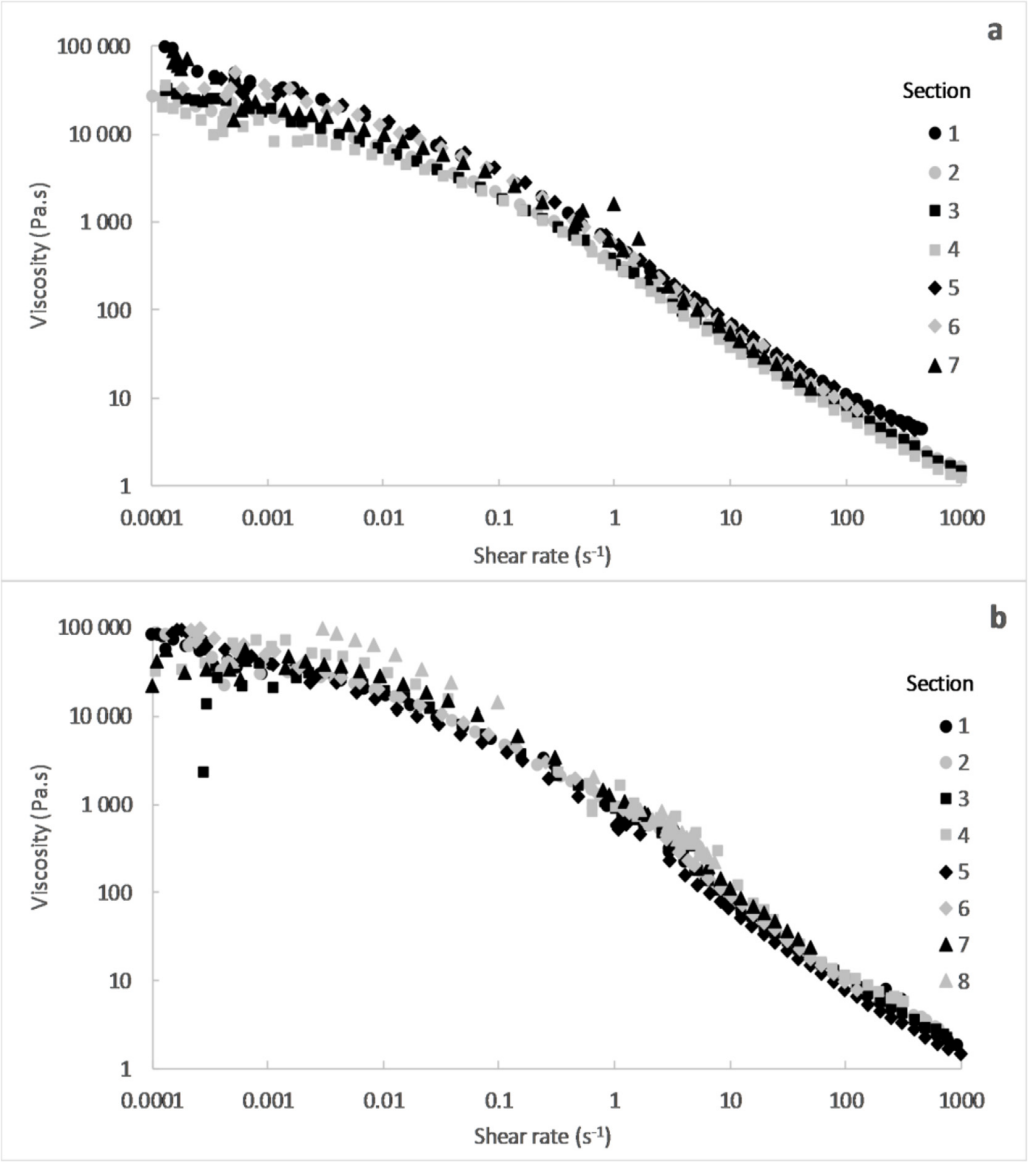
Viscosity versus shear rate for the samples from the different sections of pit 1 (a) and pit 2 (b).

The viscosity versus shear rate was plotted for each section of pit 1 and 2. Fig. 7 presents the compiled viscosity curves for each pit based upon averaged data for the samples taken at each pit section. Note that no measurement could be performed for the sample representing section 8 of pit 1, as no flow could be induced during the experiments.

The samples from the different sections of pit 1 and 2 exhibited a similar shear thinning behaviour. Some scattering could be observed between the different samples below a shear rate of 0.001 s^−1^, which could be due to the low precision of the measurements at low shear rate. Above 0.001 s^−1^, the viscosities of the samples were in the same order magnitude at a given shear rate.

The yield stress were determined in the same way as described in section 3.1.2. The results are summarized in Table 1. It can be seen that, for each pit, the yield stress of the sludge from the different sections were in the same order of magnitude. The yield stress varied between 300 and 600 Pa for pit 1 and between 500 and 1000 Pa for pit 2, without showing any particular trend with respect to the position within the pit.

**Table 1:** Yield stress for the samples from the different sections of pit 1 and 2.

	Pit 1	Pit 2
Section 1	500 Pa	800 Pa
Section 2	300 Pa	1000 Pa
Section 3	400 Pa	1000 Pa
Section 4	300 Pa	600 Pa
Section 5	600 Pa	500 Pa
Section 6	600 Pa	900 Pa
Section 7	600 Pa	1000 Pa
Section 8	–	900 Pa

No significant differences on the rheological properties were observed for the sludge from the different sections of pit 1 and 2. The only exception was the sample from section 8 of pit 1, for which it was not possible to initiate a flow during the experiments in the rheometer. This could be due to the low moisture content of this sample that compromised its ability to flow.

#### 3.2.3 Comparison between the different pits

**Fig. 8:**
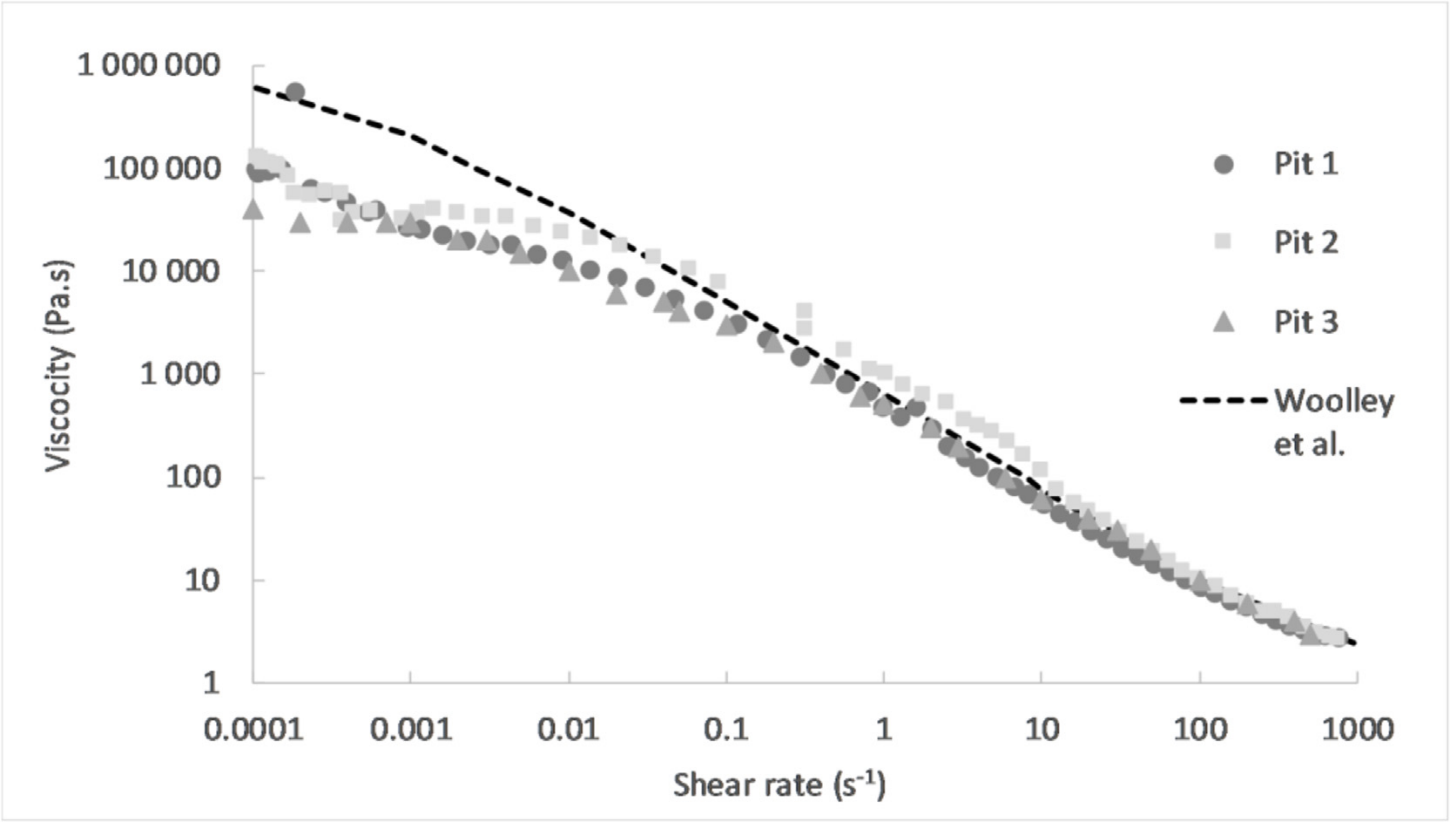
Viscosity versus shear rate for the average sludge from pit 1, pit 2 and pit 3, and for fresh faeces with a moisture content of 80.2% (Woolley et al., 2014b).

The viscosity curves from the different sections were averaged for pit 1 and pit 2, and were then compared to the curve from pit 3 in Fig. 8. The results obtained by Woolley et al. (2014b), for fresh faeces with a moisture content similar the faecal sludge samples (80.2%), were included in the graph.

In overall, the curves from pit 1, 2 and 3 looked similar in the studied shear rate interval. The viscosities of pit 2 was slightly higher in the shear rate interval from 0.001 to 10 s^−1^. Beyond this point, the three pits had approximately the same viscosity. The average yield stress of the sludge from the different pits were in the same order of magnitude, with value of 500, 700 and 400 Pa for pit 1, 2 and 3 respectively. So then, the faecal sludge from the three pits exhibited globally similar rheological characteristics with only some minor differences.

A similar rheology behaviour was observed between the fresh faeces and faecal sludge above a shear rate of 0.01 s^−1^. In contrast, the viscosity of fresh faeces was considerably higher at shear rates lower than 0.01 s^−1^. It was noted that this difference occurred before the yield stress of the sludge samples was reached. This suggests that the fresh faeces had viscoelastic properties different than the faecal sludge from pit latrines.

#### 3.2.4 General discussion

This study demonstrated that the rheology of faecal sludge was related to the moisture content. Thereby, the yields stress and viscosity tended to decrease as the moisture content was augmented. Note that no relationship between the ash content and the rheological characteristics could be established in this study.

Most of the samples from pit 1, 2 and 3 had a similar moisture content around 80% and hence they exhibited similar viscosities. The faecal sludge from section 8 of pit 1, with considerable lower moisture content, presented a different behaviour and was not able to flow during the rheometer experiments. This sample did not exhibit the behaviour of a shear thinning fluid, on the contrary to the rest of the samples.

In the eThekwini Municipality, the moisture content of faecal sludge from VIP latrines mostly varies around 80% (Zuma et al., 2015), so it can be expected a low variability of sludge viscosity for pit emptying and treatment process applications. Nonetheless, Zuma et al. (2015) also observed that the sludge at the bottom of some pits can have lower moisture content compared to the average, which will considerably impact the rheological properties and thus it will influence the way that the sludge will have to be handled. In fact, the sludge at the bottom of the pit is the most aged, so the possibility to be biodegraded is more important compared to the rest of the sludge. The degraded faecal sludge can lose its ability to flow, as seen for the sample from section 8 of pit 1.

### 3.3 Faecal sludge pumping

Calculations were performed in order to estimate the pumping requirements during pit emptying, based on the criteria from the OI project, as described with more detail in section 2.3. The objective is to provide an indication of how much water should be added for easy workability of faecal sludge and so to facilitate pit emptying. Assuming an initial faecal sludge moisture content of 75%, Table 2 displays the amount of water to add to increase the moisture content to a given value, as well as the consequent head and power required for pumping. The estimated time to empty a pit with a supposed volume of 2000 L was included in Table 2.

**Table 2:** Head and energy requirements for the pumping of faecal sludge with different moisture contents after the addition of water into the pit.

Parameters	Moisture content %				
	75	80	85	90	95
Water added (L)	0	700	1867	4200	11,200
Pit emptying time (min)	11	15	21	34	73
Head (m)	537	84	16	3	1
Power (W)	22,000	3500	600	100	40

For faecal sludge with a moisture content below 80%, pumping would be not technically possible or would require high capacity slurry pumps, which is not a feasible option in the context of pit emptying. The addition of water would drastically decrease the pumping requirements. By increasing the moisture content from 75 to 95%, the head and power for sludge pumping would be lowered by a factor of 100 to 1000. For faecal sludge with moisture content higher than 85%, pit emptying could be performed at head and power lower than 20 m and 1 KW respectively, which can be achieved with small pump units.

In the other hand, the addition of water during pit emptying presents drawbacks. For example, the amount of water in the sludge has to be triplicated in order to increase the moisture content from 75% wet basis (3 g moisture/g dry solid) to 90% wet basis (9 g moisture/g dry solid). In consequence, pit emptying time will be also triplicated, which will considerably lower the number of pits that could be emptied in a day. Moreover, the pit emptying contractor would have to bring considerable amounts of water to the pit, namely 4200 L that represents the double of the volume of the pit to empty in the aforementioned example. This will add to the cost of emptying and will make more difficult the operation of mixing the sludge with water. Besides, the addition of water can negatively affect the performance of upstream process, such as drying. To increaser the moisture content of the sludge up to 95%, excessively large quantities of water will have to be employed (11,200 L) and one pit will take more than one hour to be emptied.

A compromise has to be then made between water addition into the pit and the ability of the sludge to be pumped. In the example illustrated in Table 2, the increase of the sludge moisture content up to 85% seems a reasonable option for pit emptying. Below a moisture content of 80%, the pumping requirements might be too high and, above a moisture content of 90%, pit emptying will require unpractical amounts of water and time.

## 4 Conclusions

In this study, the faecal sludge from VIP latrines demonstrated a shear thinning behaviour, i.e. a decrease of viscosity at increasing shear rate. The presence of a yield stress suggests that the sludge has an elastic resistance to overcome in order to start to flow. Compared to fresh faeces, faecal sludge has similar rheological characteristics while flowing.

The rheological behaviour of faecal sludge depends considerably on the moisture content. Therefore, samples with similar moisture content showed a comparable viscosity, regardless the position within the pit or the pit latrine from where they were collected. This suggests that, for pits with the same moisture content, the same operating settings could be employed during mechanised pit emptying. Nonetheless, an important aspect to consider is the relative low moisture content that faecal sludge can sometimes attain at the bottom of the pit, due to the dehydration of the sludge related to its aging. This would alter considerably the rheological properties of the sludge and compromise its flowability.

Mechanised pit emptying is a challenging practice due to the difficulty in initiating flow of the faecal sludge, apart from other issues as the usual poor access to the pits and presence of trash. The addition of water can assist to pit emptying, as it increases the flowability of faecal sludge by reducing the viscosity and shear yield. As a consequence, the head and energy requirements would be drastically lowered. However, water addition elongates the pit emptying time, makes the operation more complicated and is not suitable for certain upstream processes, as for example drying. Therefore, for each specific case, an assessment has to be made in order to find the compromise between facilitating pumping and adding water into the pit.

Future investigations should include other aspects, such as the temperature and the presence of trash. Indeed, the ambient temperature in certain regions of the planet, considerably higher than in the case of this study, could alter the faecal sludge rheological properties. Moreover, the pits usually present high amounts of trash that could modify the flowing behaviour of the sludge.

## References

[bib1] Amiri H., Arrabhosseini A., Kianmehr M.H. (2012). Determination of some rheological properties of cow manure using a shear vane. Egypt. Acad. J. Biol. Sci..

[bib2] APHA (2012). Standard Methods for the Examination of Water and Wastewater.

[bib3] Bakare B.F., Foxon K.M., Brouckaert C.J., Buckley C.A. (2012). Variation in VIP latrine sludge contents. WaterSA.

[bib4] Belcher D., Foutch G.L., Smay J., Archer C., Buckley C.A. (2015). Viscous heating effect on deactivation of helminth eggs in ventilated improved pit sludge. Water Sci. Technol..

[bib5] BMGF (2012). Reinvent the Toilet Challenge. http://www.gatesfoundation.org/What-We-Do/Global-Development/Reinvent-the-Toilet-Challenge.

[bib6] Brown N.P., Heywood N.I. (1991). Slurry Handling: Design of Solid-liquid Systems.

[bib7] Cairncross S., Hunt C., Boisson S., Bostoen K., Curtis V., Fung I.C.H., Schmidt W.-P. (2010). Water, sanitation and hygiene for the prevention of diarrhoea. Int. J. Epidemiol..

[bib8] Chilton R.A., Stainsby R. (1998). Pressure loss equations for laminar and turbulent non-Newtonian pipe flow. J. Hydraul. Eng..

[bib9] Dodane P.H., Ronteltap M. (2014). Unplanted drying beds. Faecal Sludge Management: Systems Approach for Implementation and Operation.

[bib10] El-Mashad H.M., van Loon W.K.P., Zeeman G., Bot G.P.A. (2005). Rheological properties of dairy cattle manure. Bioresour. Technol..

[bib11] Ghafoori E., Flynn P.C., Feddes J.J. (2007). Pipeline vs. truck transport of beef cattle manure. Biomass Bioenergy.

[bib12] Habitat U.N. (2008). The State of African Cities 2008.

[bib13] Jain N. (2011). Getting Africa to Meet the Sanitation MDG: Lessons from Rwanda.

[bib14] Metzner A.B., Reed J.C. (1955). Flow of non-Newtonian fluids—correlation of the laminar, transition, and turbulent-flow regions. AIChE J..

[bib15] Mikhael G., Robbins D.M., Ramsay J.E., Mbéguéré M. (2014). Methods and means for collection and transport of faecal sludge. Faecal Sludge Management: Systems Approach for Implementation and Operation.

[bib16] Niwagaba C.B., Mbéguéré M., Strande L. (2014). Faecal sludge quantification, characterization and treatment objectives. Faecal Sludge Management: Systems Approach for Implementation and Operation.

[bib17] Nwaneri C.F. (2009). Physico-chemical Characteristics and Biodegradability of Contents of Ventilated Improved Pit Latrines (VIPs) in EThekwini Municipality (DISS).

[bib18] Nwaneri C.F., Foxon K., Bakare B.F., Buckley C. (2008). Biological degradation processes within a pit latrine. WISA 2008 Conference, Sun City.

[bib19] O'Riordan M. (2009). Investigation into Methods of Pit Latrine Emptying. Partners Dev. WRC Proj. 1745.

[bib20] Pillay S., Bhagwan J. (2014). Sanitation Research Fund of BMGF/WRC (SRFA): Knowledge-based Solutions for Onsite Dry Sanitation Challenges in Sub-saharan Africa. http://www.susana.org/en/resources/library/details/2106.

[bib21] PRG (2014). Standard Operating Procedures. http://prg.ukzn.ac.za/laboratory-facilities/standard-operating-procedures.

[bib22] Seyssiecq I., Ferrasse J.-H., Roche N. (2003). State-of-the-art: rheological characterisation of wastewater treatment sludge. Biochem. Eng. J..

[bib23] Strande L. (2014). The way forward. Faecal Sludge Management: Systems Approach for Implementation and Operation.

[bib24] Strauss M., Heinss U., Montangero A. (1999). On-site sanitation: when the pits are full–planning for resource protection in faecal sludge management. Schriftenr. des Vereins fur Wasser-, Boden-und Lufthygiene.

[bib25] Thye Y.P., Templeton M.R., Ali M. (2011). A critical review of technologies for pit latrine emptying in developing countries. Crit. Rev. Environ. Sci. Technol..

[bib26] Woolley S.M., Buckley C.A., Pocock J., Foutch G.L. (2014). Rheological modelling of fresh human faeces. J. Water, Sanit. Hyg. Dev..

[bib27] Woolley S.M., Cottingham R.S., Pocock J., Buckley C.A. (2014). Shear rheological properties of fresh human faeces with different moisture content. WaterSA.

[bib28] Zuma L., Velkushanova K., Buckley C.A. (2015). Chemical and thermal properties of dry VIP latrine sludge. WaterSA.

